# Real-Time Cerebral Vessel Segmentation in Laser Speckle Contrast Image Based on Unsupervised Domain Adaptation

**DOI:** 10.3389/fnins.2021.755198

**Published:** 2021-11-30

**Authors:** Heping Chen, Yan Shi, Bin Bo, Denghui Zhao, Peng Miao, Shanbao Tong, Chunliang Wang

**Affiliations:** ^1^School of Biomedical Engineering, Shanghai Jiao Tong University, Shanghai, China; ^2^School of Technology and Health, KTH Royal Institute of Technology, Stockholm, Sweden

**Keywords:** laser speckle contrast imaging, vessel segmentation, CycleGAN, domain adaptation, blood flow imaging

## Abstract

Laser speckle contrast imaging (LSCI) is a full-field, high spatiotemporal resolution and low-cost optical technique for measuring blood flow, which has been successfully used for neurovascular imaging. However, due to the low signal–noise ratio and the relatively small sizes, segmenting the cerebral vessels in LSCI has always been a technical challenge. Recently, deep learning has shown its advantages in vascular segmentation. Nonetheless, ground truth by manual labeling is usually required for training the network, which makes it difficult to implement in practice. In this manuscript, we proposed a deep learning-based method for real-time cerebral vessel segmentation of LSCI without ground truth labels, which could be further integrated into intraoperative blood vessel imaging system. Synthetic LSCI images were obtained with a synthesis network from LSCI images and public labeled dataset of Digital Retinal Images for Vessel Extraction, which were then used to train the segmentation network. Using matching strategies to reduce the size discrepancy between retinal images and laser speckle contrast images, we could further significantly improve image synthesis and segmentation performance. In the testing LSCI images of rodent cerebral vessels, the proposed method resulted in a dice similarity coefficient of over 75%.

## Introduction

Laser speckle contrast imaging (LSCI) is based on the scattering properties of moving particles (e.g., red blood cells) in tissues (Fercher and Briers, 1981). When a coherent light beam illuminates the diffusing surface, the back-scattered lights interfere and superimpose randomly, generating bright and dark speckles (Briers et al., 2013). Full-field and high spatiotemporal flow map could be obtained with spatial laser speckle contrast analysis (s-LASCA) (Briers and Webster, 1996) or temporal laser speckle contrast analysis (t-LASCA) (Cheng et al., 2003). LSCI therefore could provide both functional and structural information of blood vessels, and it has been widely used in both clinical and biomedical researches for its merits of high resolution and low cost. So far, LSCI has mainly been used to quantitatively or qualitatively monitor the blood flow or perfusion change at a selected vessel or region of interest.

Blood vessel segmentation is of high interest in biomedical image processing, as the morphological characteristics like diameter, tortuosity, and shape of blood vessels are critical for early diagnosis, treatment planning, and evaluation (Fan et al., 2019). So far, there have been high-performance methods for blood vessel segmentation in computed tomography angiography (CTA), magnetic resonance angiography (MRA), and color fundus photography (CFP) (Moccia et al., 2018). However, due to the low signal–noise ratio and relatively much smaller sizes of the cerebral vessels, particularly in rodent animal studies, segmenting the cerebral vessels has always been challenging. In addition, real-time segmentation is another technical challenge in vascular pattern recognition, which is quite useful during intraoperative procedures. By and large, there are limited literature on the real-time blood vessel segmentation for LSCI.

Recently, deep convolutional neural network (DCNN)-based biomedical image segmentation has become increasingly popular (Soomro et al., 2019). In comparison with conventional machine learning-based segmentation that requires for human to interfere in the feature extraction, deep learning approaches take the advantages of training with a large number of images using the internal image features. The development of fully convolutional network (FCN) (Long et al., 2015) has greatly improved the vascular segmentation. Several FCN-based segmentation networks have been proposed, such as U-Net (Ronneberger et al., 2015) and SegNet (Badrinarayanan et al., 2015). However, the efficiency of deep learning models relies on the availability of a great number of labeled images, while in many clinical cases, such annotated data may be quite limited or even non-existing. Usually, manual annotation is required to create the ground truth, which, however, is quite time consuming and expensive in the cases of blood vessel segmentation. Another challenge in deep learning is its generalizability, i.e., models achieving high performance on the training data may have poor performance on the testing data. Deep learning models are more likely to achieve good outcomes on testing images from the same domain as the training data. However, its performance is compromised when there is domain shift from training data to test data due to variations in the acquisition device noise, tissue structures, etc. (Javanmardi and Tasdizen, 2018).

So far, there have been few efficient approaches for vessel segmentation in LSCI; for example, our group used the OTSU method to segment cortical arteries and veins, which was successful for segmenting the larger vessels (Zhao et al., 2014). Although DCNN could potentially solve this problem, obtaining the ground truth for laser speckle contrast images is laborious and time consuming due to the low signal–noise ratio of LSCI and the non-homogeneity of illumination, which also may degrade the performance of DCNN. In this manuscript, we propose a real-time method for cerebral vessel segmentation in LSCI based on unsupervised domain adaptation without the ground-truth labels in the target modality. First, synthetic laser speckle contrast images were obtained with a synthesis network from the unpaired images of LSCI and publicly available labeled datasets of Digital Retinal Images for Vessel Extraction (DRIVE). Then, synthetic laser speckle contrast images with corresponding ground truth of fundus images in DRIVE were used to train the segmentation network. To reduce the size mismatch between retinal images and laser speckle contrast images, we further implemented two strategies for size matching. With the same dataset, we systematically compared the LSCI vessel segmentation performances by different training methods and different size-matching strategies, in comparison with the standard OTSU’s threshold method.

## Related Work

### Image Synthesis

When there are insufficient raw training images for training the deep learning networks, domain adaptation has been a successful alternative way in biomedical image segmentation. Domain adaptation techniques usually construct synthesized images by mapping the source and target images onto a common feature space with the synthesis network.

Generative adversarial networks (GANs) have been extensively applied to image synthesis and domain adaptation (Osokin et al., 2017; Yi et al., 2019). Isola et al. (2017) proposed a *pix2pix* algorithm for image-to-image translation with conditional generative adversarial networks (CGANs), which was trained with paired source and target images from different domains. However, paired images for the same anatomical structure usually are not easy to acquire in biomedical practice, which thus greatly limits the application of *pix2pix* in practice. To solve this problem, Zhu et al. (2017) proposed a cycle-consistent GAN (CycleGAN) to be trained with unpaired images from both source and target domains. CycleGAN, thus, has been one of the most successful networks for image synthesis and domain adaptation, for example, in the applications to data augmentation of X-ray angiography (Tmenova et al., 2019), lung CT images (Sandfort et al., 2019), and retinal images (Yu et al., 2019) to enlarge the training dataset. Based on the existing synthesis networks, Armanious et al. (2020) further proposed a new GAN framework, called MedGAN, by introducing new loss functions and a new generator architecture, which could be applied to different synthesis tasks without application-specific modifications to the hyperparameters.

### Domain Adaptation for Biomedical Image Segmentation

Although so far there has been quite limited work on the segmentation of laser speckle contrast images, the extensive work on other modalities of biomedical images based on unsupervised domain adaptation and image-to-image translation could be inspiring. Chartsias et al. (2017), for example, proposed a two-stage approach for myocardial segmentation of MR images without ground truth labels. First, synthesized MR images were obtained from the publicly labeled myocardial CT images with a CycleGAN. The synthesized MR images then were used to train the myocardial MR image segmentation network with similar architecture to the U-Net. Huo et al. (2019) proposed the end-to-end SynSeg-Net for multiorgan segmentation of CT images without manual labeling, by connecting the synthesis network (CycleGAN) and segmentation network (nine block ResNet) with a one-step training strategy. Different from Agisilaos’ two-stage approach, the segmentation loss in SynSeg-Net was backward propagated through the whole network other than only optimizing the segmentation network so that the two networks were jointly trained in an end-to-end framework. Zhang et al. (2018) also proposed an end-to-end framework called TD-GAN for multiorgan segmentation of X-ray images, which incorporated a pretrained segmentation network (DI2I) with a modified CycleGAN. CycleGAN network has also been well developed to facilitate different applications in practice. For example, in order to eliminate the geometric distortion in cross-modality synthesis, Cai et al. (2019) proposed a cycle- and shape-consistent GAN for multiorgan segmentation, which ensured consistent anatomical structures in synthetic images by introducing a novel shape-consistency loss. Chen et al. (2018) proposed a semantic-aware CycleGAN named SeUDA for chest X-ray segmentation, which preserved more structural information during image transformation by embedding a nested adversarial learning in semantic label space. Jiang et al. (2018) proposed a tumor-aware CycleGAN for CT to MRI translation and lung cancer segmentation, which could better preserve tumor structures in synthetic images by introducing a novel target-specific loss called tumor-aware loss.

## Materials and Methods

### Datasets

**FIGURE 1:**
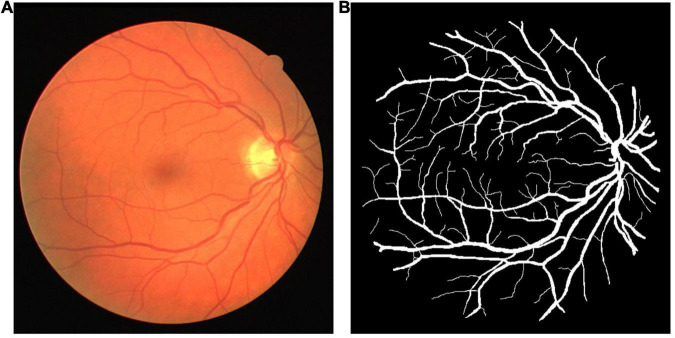
**(A)** A sample of fundus image and **(B)** its ground truth from the digital retinal images for vessel extraction (DRIVE) dataset.

**FIGURE 2:**
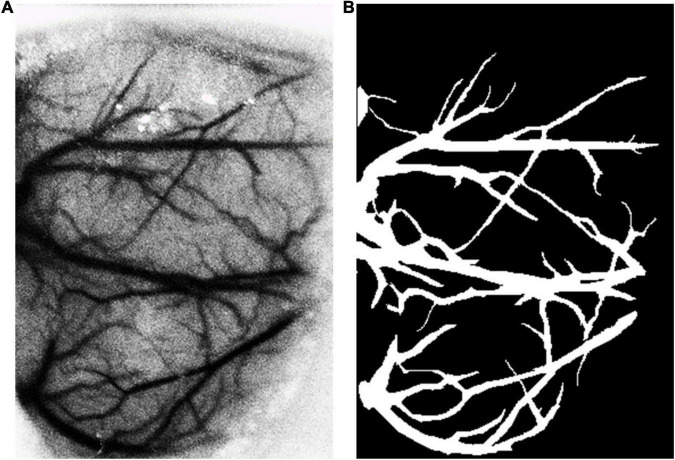
**(A)** A sample of laser speckle contrast image and **(B)** its ground truth by manual labeling.

The DRIVE dataset (Staal et al., 2004) was used as the source dataset to synthesize the labeled LSCI images and train the vessel segmentation network for its public availability, high signal–noise ratio, and well-recognized labeling as the ground truth. DRIVE includes 40 digital fundus images captured by a Canon CR5 3CCD camera with a resolution of 584 × 565 pixels. DRIVE has been one of the most popular datasets for retinal vascular segmentation. A sample of fundus image and the corresponding ground truth are illustrated in Figure 1. The laser speckle contrast images to be segmented were the target dataset, which were selected from the animal experiments for our previous studies (Bo et al., 2018, 2020), including 140 cropped images with a resolution of 400 × 280 pixels. The laser speckle images were collected from animal experiments. The experimental protocols were approved by the Institutional Animal Care and Use Committee (IACUC) of Shanghai Jiao Tong University. The images were captured by a CCD camera (DCU224M, Thorlabs, Newton, NJ, United States) during cerebral blood flow (CBF) monitoring in rat stroke model. The 140 images were randomly split into training images and testing images with the proportions of 80%:20% or 112 and 28 images, respectively, in this case. The testing images were manually segmented by three trained individuals independently, and the final ground truth was generated using a majority voting strategy. A sample of laser speckle contrast image and the corresponding ground truth are shown in Figure 2.

### Image Preprocessing

Image preprocessing techniques included grayscale conversion, image normalization, and contrast limited adaptive histogram equalization (CLAHE). Gamma correction was adopted to enhance the contrast and reduce the noises in both source and target domain images.

### Data Augmentation

A large number of images are usually required to train the DCNNs. When the number of training images was limited, data augmentation was often used to reduce the risk of overfitting and improve the network performance. In this study, we used an augmentation strategy called patch extraction. Six hundred forty patches of 256 × 256 pixels were extracted from source domain images, and another 896 patches of the same size were extracted from target domain images.

### Size Matching of Source and Target Domain

Considering the fact that the sizes of the most retinal vessels were much smaller than the cerebral vessels of rats in our case, we adopted size matching processing between source and target domain images before training the synthesis network. The same strategy was applied to the fundus manual labels for training the segmentation network to guarantee the consistency of the vessel sizes. Two matching approaches were adopted.

#### Size Matching by Vessel Dilation

The first approach for size matching method is based on vessel dilation, as defined by Eq. 1, which aims to scale the diameter of the retinal vessels by a morphological transformation,


(1) $$X⊕K=\underset{k\in K}{\cup }X_{k},$$

where *X* is the set of Euclidean coordinates corresponding to the source images, *K* is the set of coordinates for the structuring element, also called kernel, which is the basic operator in morphology, and *X*_*k*_ is the translation of *X* by *k*. For our dataset, we used a 3 × 3 structuring element with connectivity 1, that is,


(2) $$K=\left[\begin{array}{ccc} 0 & 1 & 0\\ 1 & 1 & 1\\ 0 & 1 & 0 \end{array}\right].$$

**FIGURE 3:**
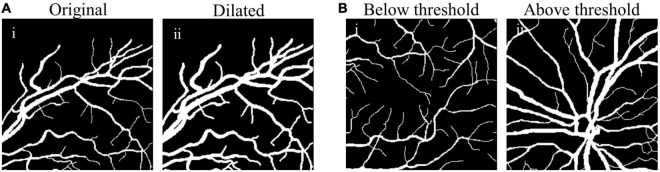
Examples of size-matching strategies in source domain. **(A)** The original fundus image **(A-i)** was transformed to the dilated image **(A-ii)** after the size matching method by vessel dilation (vdSM), and **(B)** in the size matching method by patches selection (psSM), the fundus image with the proportion of vessel pixels below threshold **(B-i)** was removed from the source domain, while that with more thick vessels **(B-ii)** was reserved.

The comparison between original and dilated fundus images is shown in Figure 3A, showing clearly enlarged vessel diameter after the size matching (Figures 3A-i vs. A-ii).

#### Size Matching by Patches Selection

Alternatively, we delicately selected those source patches with a significant proportion of large vessels to train the image synthesis network by controlling the ratio of vascular pixels to non-vascular pixels:


(3) $$\frac{N_{p}}{N_{t}}>th,$$

where *N*_*p*_ denotes the number of vascular pixels, and *N*_*t*_ denotes the number of total pixels in the source domain patch. Only those patches satisfying (3) will be used for training the synthesis network, e.g., *th* value of 0.13 was set empirically (Figure 3B).

### Network Architecture

**FIGURE 4:**
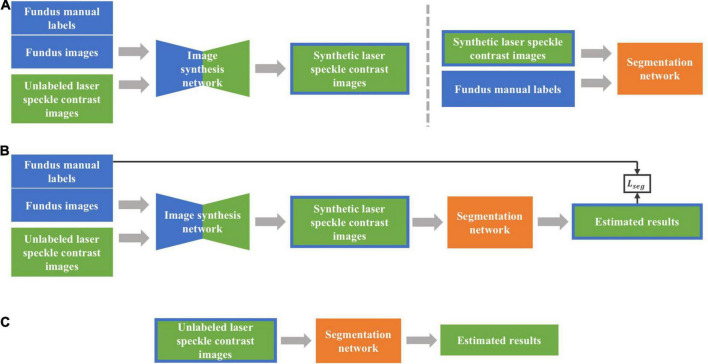
The overall framework of the proposed method. **(A)** Two-stage training approach. First, synthetic laser speckle contrast images were obtained by training an image synthesis network with labeled fundus images and unlabeled laser speckle contrast images. Then, the synthetic laser speckle contrast images and fundus manual labels were used to train a segmentation network. **(B)** End-to-end training approach. The image synthesis network and segmentation network were jointly trained, and the obtained segmentation loss was backward propagated to optimize the two networks. **(C)** Testing stage. The trained segmentation network was applied to unlabeled laser speckle contrast images to obtain the segmentation results.

Figure 4 shows the architecture of the networks to be used, including the image synthesis network and image segmentation network. Two types of training strategies, i.e., two-stage and end-to-end trainings, were implemented, respectively. Intuitively, the fundus images should be directly used as the source domain dataset with their labels only used in segmentation network, which, however, presented poor performances due to the low contrast ratio after transformed into grayscale images. Therefore, we trained both synthesis and segmentation networks with fundus manual labels. To distinguish the training images in two networks, we call the synthesis network training images as fundus images hereafter instead.

#### Two-Stage Training

In two-stage training, synthetic laser speckle contrast images were obtained by synthesis network trained with manually labeled fundus images and unlabeled laser speckle contrast images. Then, the synthetic laser speckle contrast images and the corresponding fundus labels were further used to train the segmentation network, which would be applied to real laser speckle contrast images to identify the blood vessels.

**FIGURE 5:**
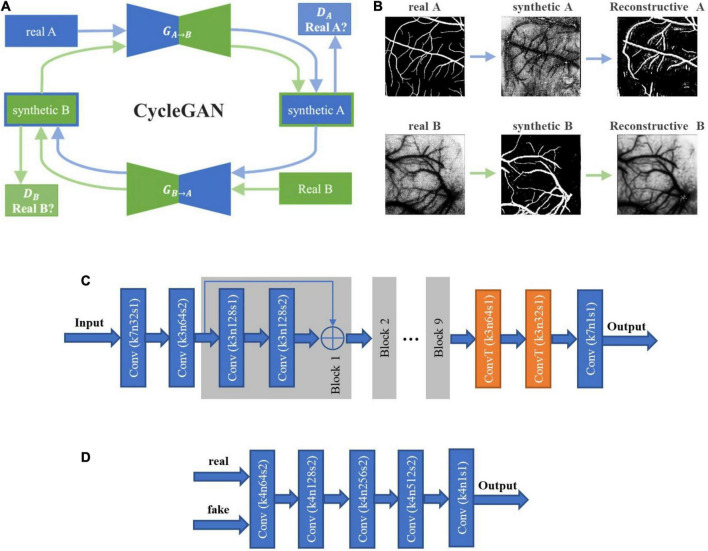
The architecture of cycle-consistent GAN (CycleGAN) and the image flow. **(A)** CycleGAN contains two generators and two discriminators trained in an adversarial way, where the architectures of generator and discriminator are shown in Panels **(C,D)**. The first generator *G*_*A→B*_ transforms images from domain A to domain B, and the discriminator *D*_*A*_ is trained to distinguish synthetic images from real images. The objective is to make the discriminator unable to discriminate between real and synthetic data. Similarly, the second generator *G*_*B→A*_ transforms images of domain B back to domain A, and the discriminator *D*_*B*_ is trained to distinguish synthetic images from real images. The synthetic images should also be able to be transformed back to the original domain by the other generator, and the objective is to minimize the difference between the transformed images and the originals. **(B)** The image flow of CycleGAN. The upper panels represent the path from domain A to domain B, and the lower ones show the reverse path. In this study, domains A and B correspond to the fundus images and the laser speckle images, respectively. **(C)** The architecture of the generators *G*_*A→B*_ and *G*_*B→A*_. **(D)** The architecture of the discriminators *D*_*A*_ and *D*_*B*_.

The image synthesis network was built with CycleGAN (Zhu et al., 2017), as shown in Figure 5. The nine block ResNet was employed as the two generators *G*_*A→B*_ and *G*_*B→A*_, and the PatchGAN was employed as the two discriminators *D*_*A*_ and *D*_*B*_. The objective to the image synthesis network training is to minimize the loss function:


(4) $$\begin{array}{c} L\left(G_{A\to B},G_{B\to A},D_{A},D_{B}\right)=\lambda_{1}L_{GAN}\left(G_{A\to B},D_{B}\right)+\lambda_{2}L_{GAN}\\ \left(G_{B\to A},D_{A}\right)+\lambda_{3}L_{cyc}\left(G_{A\to B},G_{B\to A}\right) \end{array}$$

Where λ_1_, λ_2_, and λ_3_ control the weights of adversarial loss and cycle consistency loss.

The image segmentation network was built with U-Net (Ronneberger et al., 2015), and the Dice Coefficient loss was employed as the loss function to be minimized during the training:


(5) $$L_{dice}=1-\frac{2\sum_{i}^{N}p_{i}g_{i}}{\sum_{i}^{N}p_{i}^{2}+\sum_{i}^{N}g_{i}^{2}},$$

where *p*_*i*_ denotes the predicted segmentation binary result of each pixel, dotes the ground truth, and *N* is the number of pixels.

#### End-to-End Training

With the end-to-end training strategy, the image synthesis network and the image segmentation network were jointly trained, so that the two networks could be optimized by a single loss function. The synthetic network and segmentation network were the same as those in two-stage training, with the input of which were also fundus images and laser speckle contrast images. The full loss function is expressed as the weighted sum of adversarial, cycle consistency, and segmentation losses:


(6) $$\begin{array}{c} L\left(G_{A\to B},G_{B\to A},D_{A},D_{B}\right)=\lambda_{1}L_{GAN}\left(G_{A\to B},D_{B}\right)+\\ \lambda_{2}L_{GAN}\left(G_{B\to A},D_{A}\right)+\lambda_{3}L_{cyc}\left(G_{A\to B},G_{B\to A}\right)+\lambda_{4}L_{dice}. \end{array}$$

where λ_4_ controls the weight of segmentation loss.

Adam optimizer (Kingma and Ba, 2014) was used to minimize all the loss functions (Eqs 4–6) in both training strategies.

### Training and Testing

In the two-stage training approach, CycleGAN was first trained from scratch with 150 epochs with a learning rate of 0.0002 for the first two thirds of epochs and then linearly decayed to zero over the rest epochs. The loss weights in Eq. 4 were empirically set to λ_1_ = λ_2_ = 1 and λ_3_ = 10. Adam optimizer was implemented with a batch size of 1. After image synthesis, the U-Net was trained for 100 epochs with a learning rate of 0.0001. Batch normalization and a drop rate of 0.4 were used to reduce the risk of overfitting. Adam optimizer was implemented with a batch size of 8.

In the end-to-end training approach, CycleGAN and U-Net were jointly trained for 150 epochs with a learning rate of 0.0002. The loss weights in Eq. 3 were empirically set to λ_1_ = λ_2_ = λ_4_ = 1, and λ_3_ = 10.

In the testing stage, the trained segmentation network (U-Net) was employed to real laser speckle contrast images. Patches (80 × 80 pixels) were extracted from the testing images and were up-sampled to 256 × 256 pixels for further segmentation. The output patches of U-Net were down-sampled to 80 × 80 pixels again to reconstruct the segmented laser speckle contrast images.

The training and testing were carried out on a Windows10 PC with a NVIDIA GeForce RTX 2080Ti GPU (11 GB memory) and CUDA 9.0 runtime library. The codes of networks were implemented in Python 3.6 using *Keras*.

### Evaluation Metrics

The dice similarity coefficient (DSC), precision, sensitivity, and specificity were employed to evaluate the segmentation performances as in Eqs 7–10. They were calculated from true positive (TP), true negative (TN), false positive (FP), and false negative (FP), based on the pixel-wise comparison of predicted results with the ground truth.


(7) $$DSC=\frac{2TP}{FP+2TP+FN},$$

(8) $$precision=\frac{TP}{TP+FP},$$

(9) $$sensitivity=\frac{TP}{TP+FN},$$

(10) $$specificity=\frac{TN}{TN+FP}.$$

## Experimental Design and Results

### Experimental Design

The following six different configurations of the segmentation network were implemented and tested, and their performances were compared.

(a) OTSU’s threshold segmentation. OTSU aims to separate the original image into foreground and background by setting the threshold adaptively through maximizing the between-class variance.
(b) Two-stage training without size matching (2StepOnly).
(c) Two-stage training using the size matching approach of vdSM (2Step_vdSM);
(d) Two-stage training using the size matching approach of psSM (2Step_psSM);
(e) End-to-end training using the size matching approach of vdSM (E2E_vdSM);
(f) End-to-end training using the size matching approach of psSM (E2E_psSM).

### Results

#### Image Synthesis Results

**FIGURE 6:**
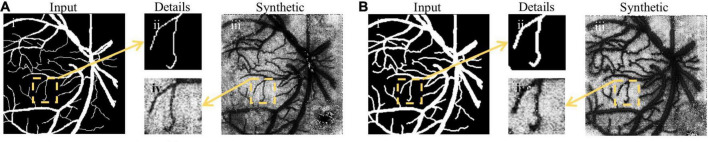
Examples of image synthesis. **(A)** Image synthesis result without size-matching; **(B)** image synthesis result with vdSM. Columns (from left to right) indicate the input fundus image, the comparison of small vessel details, and the synthetic laser speckle contrast image, respectively.

An example of image synthesis is shown in Figure 6. It is noted that the synthetized laser speckle contrast image was very alike the real one. The cosine similarity between the fundus images and the binary synthetic images is used for evaluating the synthesis performance. The average similarity coefficients are 0.918, 0.943, and 0.933 in 2StepOnly, 2Step_vdSM, and 2Step_psSM strategies, respectively. Synthetic images maintain most morphological information well, e.g., vessel location, curvature, and capillaries, etc. Besides, the consistency of the vessel diameter between the input and synthetic data is measured by the ratio of the vessel pixel proportion in two groups of images. Without size matching, the image transform resulted in 1.695 × increase in vessel diameter in the synthesized images (Figures 6A-ii vs. A-iv), which would definitely further affect the following segmentation and accounts for the lower similarity coefficient. After size matching, the vessel diameters in the synthetic and original fundus images were comparable with the ratio of 1.013 (Figure 6B), which proves the validity and necessity of the size-matching method.

#### Image Segmentation Results

**FIGURE 7:**
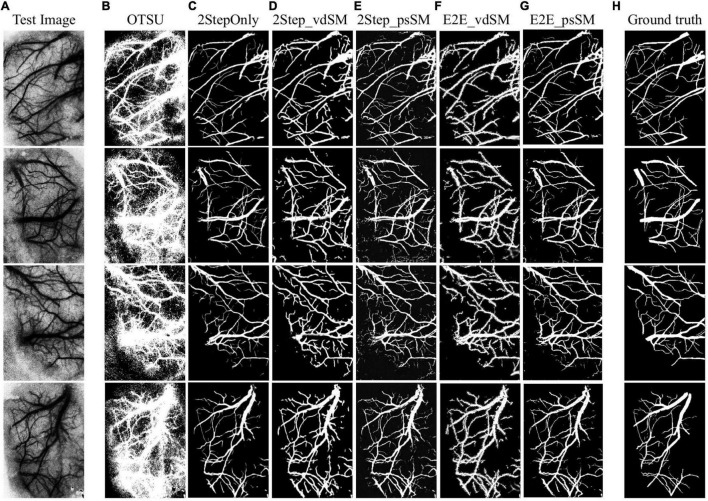
Examples of laser speckle contrast image segmentation. **(A)** Raw images. Segmentation results from: **(B)** OTSU’s threshold segmentation, **(C)** two-stage training without size matching, **(D)** two-stage training with vdSM, **(E)** two-stage training with psSM, **(F)** end-to-end training with vdSM, and **(G)** end-to-end training with psSM. **(H)** Ground truth.

Figure 7 shows the samples of segmented laser speckle contrast images (Figure 7A) corresponding to the six models as described in **Experimental Design** (Figures 7B–G), and the ground truth (Figure 7H). Table 1 summarizes the performances of the six models on the 28 testing images. The segmentation is about 10 fps when the parallel GPU calculation was adopted.

**TABLE 1:** Quantitative evaluation results for the testing images.

Model	Quantitative results (mean ± SEM)			
	DSC (%)	Precision (%)	Sensitivity (%)	Specificity (%)
OTSU	54.24 ± 6.68	37.96 ± 6.71	97.33 ± 1.76	58.99 ± 6.34
2StepOnly	70.77 ± 1.32	**94.71 ± 0.45**	51.68 ± 1.22	**99.29 ± 0.05**
2Step_vdSM	**75.81 ± 1.26**	90.29 ± 0.82	**61.98 ± 1.80**	98.34 ± 0.14
2Step_psSM	73.00 ± 0.47	83.39 ± 0.95	58.03 ± 1.10	97.12 ± 0.15
E2E_vdSM	70.60 ± 0.55	80.56 ± 1.12	60.65 ± 0.97	96.35 ± 0.19
E2E_psSM	70.18 ± 0.71	87.99 ± 0.83	58.02 ± 1.20	98.02 ± 0.13

*The best performances by the deep learning methods are highlighted.*

The standard OTSU’s threshold segmentation shows the poorest performances due to the high speckle noise and low resolution of laser speckle contrast images. In contrast, any of the domain adaptation methods remarkably outperformed OTSU. Compared with 2StepOnly, those models using vessel size matching processing (vdSM or psSM) could further improve the segmentation. In aspect of DSC, two-stage training approaches (2Step_vdSM and 2Step_psSM) had better quantitative performance than the end-to-end training approaches (E2E_vdSM and E2E_psSM).

## Conclusion and Discussion

This manuscript presents a real-time laser speckle contrast image segmentation without using ground truth labels in the target modality. Using unsupervised domain adaptation and size matching between fundus images and laser speckle contrast images, we achieved good segmentation performance for the test dataset. The proposed method could potentially be applied to automatic blood vessel segmentation for LSCI, for example, in an auxiliary system for surgical operations.

With the selected source domain images, the proposed method could also be extended to other imaging modalities like laser Doppler imaging (LDI), optical intrinsic imaging (OIS), and optical coherence tomography (OCT) for blood vessel segmentation. The real-time segmentation could facilitate its intraoperative applications. Table 1 shows that the traditional vessel segmentation method, OTSU’s threshold segmentation, had poor performance because the threshold is susceptible to the background noise. After domain adaptation by deep learning, e.g., 2StepOnly, the vascular network could be well segmented though with smaller vessel diameter than the ground truth, which was caused by the geometry discrepancy of the blood vessels in two domains. After further matching the vessel sizes using vdSM and psSM prior to the segmentation, the performance was significantly improved. Besides, it was also noted that the two-stage training (2Step_vdSM and 2Step_psSM) could outperformed the end-to-end training (E2E_vdSM and E2E_psSM). It might be related to the selection of intermediate synthetic results, which has an influence on the training of segmentation model. In two-stage strategy, we visually inspected all the intermediate results in aspects of the style, vessel integrity, signal–noise ratio (SNR), and contrast. We then selected relatively better ones for training the segmentation network. The lack of intervention in end-to-end strategy might lead to the inferior performance of segmentation. By all means, the difference between two-stage and end-to-end strategies deserves a further comprehensive study.

It was noted that some capillary vessels were bolder in the segmentation results after size matching processing (Figures 7D–G), which was more prominent by vdSM than psSM, in either two-stage or end-to-end training approach. In case when the capillary vessels are of interest, we need to further improve the segmentation algorithm, for example, using super-resolution algorithm to reconstruct a high-resolution image from a low-resolution one (Chen et al., 2020). Besides, for our dataset, the size matching was achieved by a structuring element with connectivity 1 in vdSM, while in practice, the connectivity number could be vessel dependent.

The segmentation speed could be further improved by optimizing the hardware and algorithm. In our experiments, for example, we used the same weight configuration for generative adversarial loss, cycle consistency loss, and segmentation loss as in Huo et al. (2019). Further investigation on the tuning hyperparameters of the model could be conducted on the cross-validation as recommended. Besides, we used nine block ResNet as the generators and PatchGAN as the discriminators for the image synthesis network adopted from the original CycleGAN manuscript (Zhu et al., 2017), and U-Net was used as the image segmentation network. A recent work by Yu et al. (2019) compared different generators for CycleGAN, including U-Net, ResNet, and ResU-Net, and demonstrated that U-Net performed better than nine block ResNet in the cases of retina image synthesis. Therefore, selecting appropriate generators and/or simplifying the network architecture with consideration on the segmentation speed and performance is also an interesting topic in practice.

A previous work by Jiang et al. (2018) included a small set of real labeled images into the synthetic images to train the segmentation network and showed that such kind of semi-supervised segmentation could further boost the segmentation accuracy. Therefore, we speculated that including a small number of labeled raw laser speckle contrast images, if available in practice, would train the networks more efficiently.

The selection of the training images for the segmentation network was subjective. Although we assessed the outputs of synthetic network in the aspect of cosine similarity, which however, was not used for selecting the intermediate results in this study, in this manuscript, we focus on the blood vessel segmentation rather than synthesis. Therefore, the image synthesis was simply visually inspected. Future study may consider to objectively select the intermediate results using the indices like image structure clustering (ISC) (Zhu et al., 2017), structure similarity (SSIM), or peak signal-to-noise-ratio (PSNR) (Sandfort et al., 2019).

## Data Availability Statement

The raw data supporting the conclusions of this article will be made available by the authors, upon a reasonable request.

## Ethics Statement

The animal study was reviewed and approved by Institutional Animal Care and Use Committee (IACUC) of Shanghai Jiao Tong University.

## Author Contributions

HC and YS did the data analysis, coding, and wrote the draft the manuscript. BB did the *in-vivo* experiment. DZ did the data labeling. PM wrote the codes for LASCA. ST and CW initiated and sponsored this study, designed the study, and finalized the writing. All authors contributed to the article and approved the submitted version.

## Conflict of Interest

The authors declare that the research was conducted in the absence of any commercial or financial relationships that could be construed as a potential conflict of interest.

## Publisher’s Note

All claims expressed in this article are solely those of the authors and do not necessarily represent those of their affiliated organizations, or those of the publisher, the editors and the reviewers. Any product that may be evaluated in this article, or claim that may be made by its manufacturer, is not guaranteed or endorsed by the publisher.
